# Crystal structure of 2-oxo-*N*′-phenyl-2*H*-chromene-3-carbohydrazide

**DOI:** 10.1107/S2056989015022495

**Published:** 2015-11-28

**Authors:** Joel T. Mague, Shaaban K. Mohamed, Mehmet Akkurt, Sabry H. H. Younes, Mustafa R. Albayati

**Affiliations:** a Department of Chemistry, Tulane University, New Orleans, LA 70118, USA; b Chemistry and Environmental Division, Manchester Metropolitan University, Manchester M1 5GD, England; c Chemistry Department, Faculty of Science, Minia University, 61519 El-Minia, Egypt; d Department of Physics, Faculty of Sciences, Erciyes University, 38039 Kayseri, Turkey; e Chemistry Department, Faculty of Science, Sohag University, 82524 Sohag, Egypt; f Kirkuk University, College of Science, Department of Chemistry, Kirkuk, Iraq

**Keywords:** crystal structure, coumarins, bio-activity, coumarin scaffold compounds

## Abstract

In the title compound, C_16_H_12_N_2_O_3_, the 2*H*-chromene moiety is essentially planar, with an r.m.s. deviation of the nine constituent atoms from the mean plane of 0.0093 Å, and makes a dihedral angle of 76.84 (3)° with the pendant phenyl ring. An intra­molecular N—H⋯O hydrogen bond helps to determine the conformation of the side chain. In the crystal, N—H⋯O and N—H⋯N hydrogen bonds link the mol­ecules, forming [100] chains.

## Related literature   

For synthesis and bio-activity of coumarin scaffold compounds, see: Shivashankar *et al.* (2008*a* ▸,*b* ▸, 2009 ▸); Bansal *et al.* (2013 ▸); Jacquot *et al.* (2007 ▸); Bhavsar *et al.* (2011 ▸).
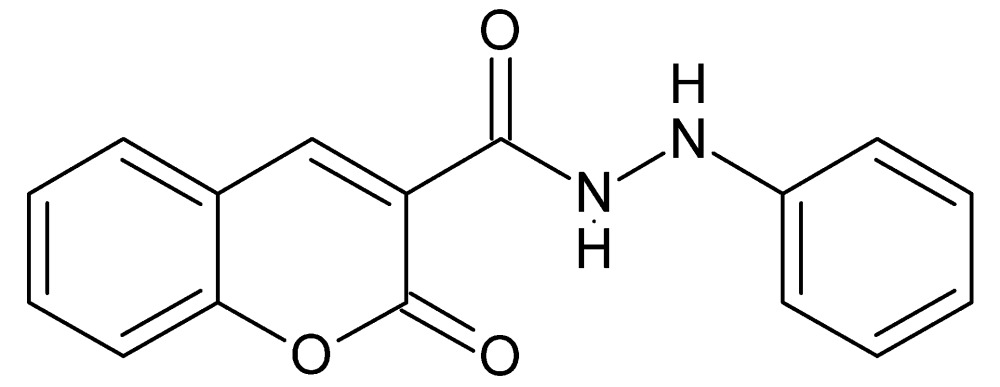


## Experimental   

### Crystal data  

C_16_H_12_N_2_O_3_
*M* *_r_* = 280.28
Triclinic, 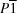

*a* = 6.6508 (2) Å
*b* = 8.3906 (3) Å
*c* = 11.6388 (4) Å
α = 96.504 (2)°
β = 95.614 (2)°
γ = 94.757 (2)°
*V* = 639.31 (4) Å^3^
*Z* = 2
Cu *K*α radiation
μ = 0.85 mm^−1^
*T* = 150 K
0.19 × 0.13 × 0.05 mm

### Data collection  

Bruker D8 VENTURE PHOTON 100 CMOS diffractometer
Absorption correction: multi-scan (*SADABS*; Bruker, 2015 ▸) *T*_min_ = 0.88, *T*_max_ = 0.96
4865 measured reflections
2371 independent reflections
2121 reflections with *I* > 2σ(*I*)
*R*_int_ = 0.023

### Refinement  

*R*[*F* ^2^ > 2σ(*F* ^2^)] = 0.037
*wR*(*F* ^2^) = 0.107
*S* = 1.06
2371 reflections
199 parameters
H atoms treated by a mixture of independent and constrained refinement
Δρ_max_ = 0.22 e Å^−3^
Δρ_min_ = −0.21 e Å^−3^

### 

Data collection: *APEX2* (Bruker, 2015 ▸); cell refinement: *SAINT* (Bruker, 2015 ▸); data reduction: *SAINT*; program(s) used to solve structure: *SHELXT* (Sheldrick, 2015*a* ▸); program(s) used to refine structure: *SHELXL2014* (Sheldrick, 2015*b* ▸); molecular graphics: *DIAMOND* (Brandenburg & Putz, 2012 ▸); software used to prepare material for publication: *SHELXTL* (Sheldrick, 2008 ▸).

## Supplementary Material

Crystal structure: contains datablock(s) global, I. DOI: 10.1107/S2056989015022495/ff2144sup1.cif

Structure factors: contains datablock(s) I. DOI: 10.1107/S2056989015022495/ff2144Isup2.hkl

Supporting information file. DOI: 10.1107/S2056989015022495/ff2144Isup3.cml

**Fig. 1.**
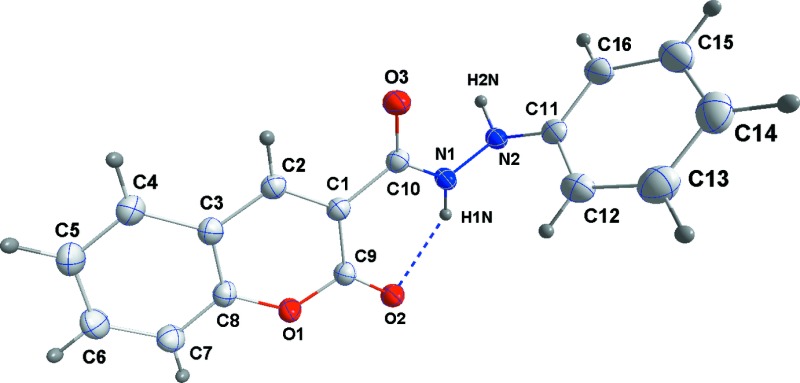
The title molecule with labeling scheme and 50% probability ellipsoids. The intramolecular N—H···O hydrogen bond is shown by a dotted line.

. DOI: 10.1107/S2056989015022495/ff2144fig1.tif
The title mol­ecule with labeling scheme and 50% probability ellipsoids. The intra­molecular N—H⋯O hydrogen bond is shown by a dotted line.

**Fig. 2.**
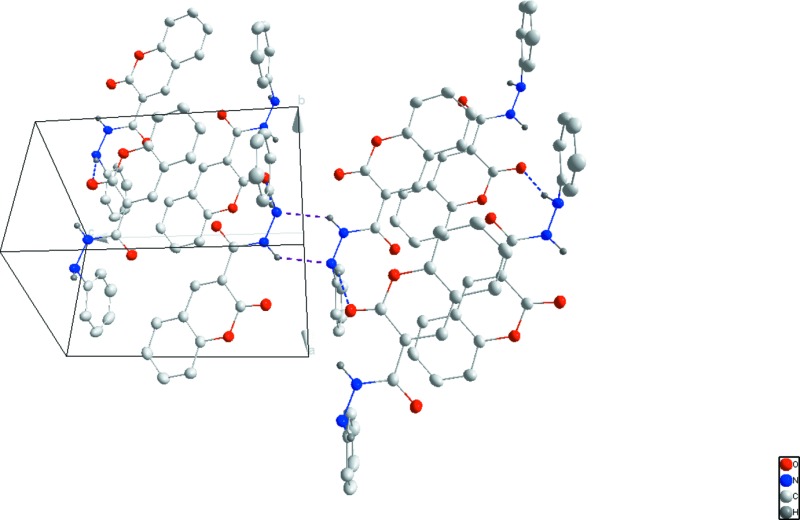
Packing viewed towards (110) with intermolecular N—H···O and N—H···N hydrogen bonds shown, respectively, as blue and purple dotted lines.

. DOI: 10.1107/S2056989015022495/ff2144fig2.tif
Packing viewed towards (110) with inter­molecular N—H⋯O and N—H⋯N hydrogen bonds shown, respectively, as blue and purple dotted lines.

CCDC reference: 1438684

Additional supporting information:  crystallographic information; 3D view; checkCIF report

## Figures and Tables

**Table 1:** Hydrogen-bond geometry (Å, °)

*D*—H⋯*A*	*D*—H	H⋯*A*	*D*⋯*A*	*D*—H⋯*A*
N1—H1*N*⋯N2^i^	0.896 (18)	2.327 (18)	3.0498 (14)	137.7 (15)
N1—H1*N*⋯O2	0.896 (18)	2.112 (18)	2.7544 (13)	127.8 (15)
N2—H2*N*⋯O2^ii^	0.911 (17)	2.243 (17)	3.1358 (14)	166.3 (14)

Symmetry codes: (i) 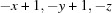; (ii) 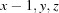.

## supplementary crystallographic information

### S1. Comment

Coumarins are known to be biologically versatile compounds possessing several biological properties. Numerous research reports have indicated the coumarin nucleus as a potential candidate for development of anti-inflammatory (Shivashankar *et al.*, 2008*a,b*; Bansal *et al.*, 2013), antibacterial (Shivashankar *et al.*, 2008*b*), antifungal (Shivashankar *et al.*, 2009), anti-cancer (Jacquot *et al.*, 2007) and anti-HIV (Bhavsar *et al.*, 2011) agents. In light of such facts, we report in this study the synthesis and crystal structure of the title compound.

The 2*H*-chromene moiety is essentially planar with an r.m.s. deviation of the nine constituent atoms from the mean plane of 0.0093 Å. The dihedral angle between this plane and that of the pendant phenyl ring is 76.84 (3)°. The conformation of the hydrazide side-chain is partially determined by an intramolecular N1—H1N···O2 hydrogen bond (Fig. 1 and Table 1). The packing is assisted by intermolecular N2—H2N···O2^i^ (i: *x* − 1, *y*, *z*) and N1—H1N···N2^ii^ (ii: −*x* + 1, −*y* + 1, −*z*) hydrogen bonds (Fig. 2 and Table 1).

### S2. Experimental

The title compound was obtained as an unexpected product from the reaction of 1-phenylpyrazolidine-3,5-dione (176 mg, 1 mmol), 2-hydroxybenzaldehyde (122 mg, 1 mmol) and *o*-toluidine (107 mg, 1 mmol). The reaction mixture was refluxed in 20 ml e thanol and monitored by TLC till completion. On cooling, the solid product was deposited, filtered off under vacuum and recrystallized from ethanol to afford colourless crystals in a sufficient quality for X-ray diffraction. *Mp* 471–473 K.

### S3. Refinement

H-atoms attached to carbon were placed in calculated positions (C—H = 0.95 Å) and included as riding contributions with isotropic displacement parameters 1.2 − 1.5 times those of the attached atoms.

### Crystal data

**Table:**

C_16_H_12_N_2_O_3_	*Z* = 2
*M**_r_* = 280.28	*F*(000) = 292
Triclinic, *P*1	*D*_x_ = 1.456 Mg m^−^^3^
*a* = 6.6508 (2) Å	Cu *K*α radiation, λ = 1.54178 Å
*b* = 8.3906 (3) Å	Cell parameters from 3784 reflections
*c* = 11.6388 (4) Å	θ = 3.9–72.2°
α = 96.504 (2)°	µ = 0.85 mm^−^^1^
β = 95.614 (2)°	*T* = 150 K
γ = 94.757 (2)°	Tablet, colourless
*V* = 639.31 (4) Å^3^	0.19 × 0.13 × 0.05 mm

### Data collection

**Table:**

Bruker D8 VENTURE PHOTON 100 CMOS diffractometer	2371 independent reflections
Radiation source: INCOATEC IµS micro–focus source	2121 reflections with *I* > 2σ(*I*)
Mirror monochromator	*R*_int_ = 0.023
Detector resolution: 10.4167 pixels mm^-1^	θ_max_ = 72.3°, θ_min_ = 3.9°
ω scans	*h* = −7→7
Absorption correction: multi-scan (*SADABS*; Bruker, 2015)	*k* = −9→10
*T*_min_ = 0.88, *T*_max_ = 0.96	*l* = −14→14
4865 measured reflections	

### Refinement

**Table:**

Refinement on *F*^2^	Secondary atom site location: difference Fourier map
Least-squares matrix: full	Hydrogen site location: mixed
*R*[*F*^2^ > 2σ(*F*^2^)] = 0.037	H atoms treated by a mixture of independent and constrained refinement
*wR*(*F*^2^) = 0.107	*w* = 1/[σ^2^(*F*_o_^2^) + (0.0632*P*)^2^ + 0.1234*P*] where *P* = (*F*_o_^2^ + 2*F*_c_^2^)/3
*S* = 1.06	(Δ/σ)_max_ < 0.001
2371 reflections	Δρ_max_ = 0.22 e Å^−^^3^
199 parameters	Δρ_min_ = −0.21 e Å^−^^3^
0 restraints	Extinction correction: *SHELXL2014* (Sheldrick 2015b), Fc^*^=kFc[1+0.001xFc^2^λ^3^/sin(2θ)]^-1/4^
Primary atom site location: structure-invariant direct methods	Extinction coefficient: 0.0129 (16)

### Special details

**Table:**

Geometry. All e.s.d.'s (except the e.s.d. in the dihedral angle between two l.s. planes) are estimated using the full covariance matrix. The cell e.s.d.'s are taken into account individually in the estimation of e.s.d.'s in distances, angles and torsion angles; correlations between e.s.d.'s in cell parameters are only used when they are defined by crystal symmetry. An approximate (isotropic) treatment of cell e.s.d.'s is used for estimating e.s.d.'s involving l.s. planes.
Refinement. Refinement of *F*^2^ against ALL reflections. The weighted *R*-factor *wR* and goodness of fit *S* are based on *F*^2^, conventional *R*-factors *R* are based on *F*, with *F* set to zero for negative *F*^2^. The threshold expression of *F*^2^ > σ(*F*^2^) is used only for calculating *R*-factors(gt) *etc*. and is not relevant to the choice of reflections for refinement. *R*-factors based on *F*^2^ are statistically about twice as large as those based on *F*, and *R*- factors based on ALL data will be even larger. H-atoms attached to carbon were placed in calculated positions (C—H = 0.95 Å) and included as riding contributions with isotropic displacement parameters 1.2 − 1.5 times those of the attached atoms.

### Fractional atomic coordinates and isotropic or equivalent isotropic displacement parameters (Å^2^)

**Table:**

	*x*	*y*	*z*	*U*_iso_*/*U*_eq_	
O1	1.05849 (13)	0.30993 (11)	0.29083 (7)	0.0271 (2)	
O2	0.92419 (13)	0.42696 (12)	0.14651 (7)	0.0299 (2)	
O3	0.43044 (13)	0.53972 (11)	0.33020 (7)	0.0292 (2)	
N1	0.52900 (16)	0.50161 (13)	0.14932 (9)	0.0256 (3)	
N2	0.35679 (16)	0.56386 (13)	0.09797 (9)	0.0245 (3)	
C1	0.73626 (18)	0.41687 (14)	0.31115 (10)	0.0220 (3)	
C2	0.74197 (18)	0.37498 (14)	0.42062 (10)	0.0233 (3)	
H2	0.6338	0.3978	0.4656	0.028*	
C3	0.90772 (18)	0.29704 (14)	0.46963 (10)	0.0240 (3)	
C4	0.9195 (2)	0.24825 (16)	0.58159 (11)	0.0275 (3)	
H4	0.8142	0.2670	0.6296	0.033*	
C5	1.0850 (2)	0.17297 (16)	0.62133 (11)	0.0300 (3)	
H5	1.0926	0.1391	0.6967	0.036*	
C6	1.2410 (2)	0.14627 (16)	0.55202 (11)	0.0305 (3)	
H6	1.3550	0.0960	0.5812	0.037*	
C7	1.2321 (2)	0.19208 (16)	0.44109 (11)	0.0295 (3)	
H7	1.3377	0.1730	0.3934	0.035*	
C8	1.06438 (19)	0.26654 (15)	0.40167 (10)	0.0243 (3)	
C9	0.90475 (18)	0.38814 (15)	0.24263 (10)	0.0236 (3)	
C10	0.55367 (18)	0.49380 (14)	0.26524 (10)	0.0229 (3)	
C11	0.35631 (19)	0.73381 (15)	0.11707 (9)	0.0246 (3)	
C12	0.5353 (2)	0.83552 (17)	0.13527 (11)	0.0324 (3)	
H12	0.6626	0.7916	0.1390	0.039*	
C13	0.5280 (2)	1.00138 (18)	0.14802 (13)	0.0401 (4)	
H13	0.6509	1.0703	0.1608	0.048*	
C14	0.3449 (2)	1.06733 (18)	0.14238 (12)	0.0390 (4)	
H14	0.3410	1.1810	0.1518	0.047*	
C15	0.1668 (2)	0.96594 (18)	0.12283 (12)	0.0367 (3)	
H15	0.0400	1.0106	0.1185	0.044*	
C16	0.1711 (2)	0.80054 (16)	0.10956 (11)	0.0300 (3)	
H16	0.0478	0.7323	0.0953	0.036*	
H2N	0.242 (3)	0.5112 (19)	0.1179 (14)	0.033 (4)*	
H1N	0.621 (3)	0.472 (2)	0.1014 (16)	0.043 (5)*	

### Atomic displacement parameters (Å^2^)

**Table:**

	*U*^11^	*U*^22^	*U*^33^	*U*^12^	*U*^13^	*U*^23^
O1	0.0260 (5)	0.0348 (5)	0.0226 (4)	0.0082 (4)	0.0054 (3)	0.0060 (4)
O2	0.0274 (5)	0.0422 (6)	0.0222 (4)	0.0063 (4)	0.0070 (3)	0.0075 (4)
O3	0.0284 (5)	0.0393 (5)	0.0221 (4)	0.0101 (4)	0.0069 (4)	0.0040 (4)
N1	0.0259 (5)	0.0337 (6)	0.0194 (5)	0.0111 (4)	0.0041 (4)	0.0048 (4)
N2	0.0230 (5)	0.0297 (6)	0.0218 (5)	0.0067 (4)	0.0027 (4)	0.0048 (4)
C1	0.0234 (6)	0.0217 (6)	0.0207 (6)	0.0020 (4)	0.0035 (5)	0.0012 (4)
C2	0.0247 (6)	0.0240 (6)	0.0210 (6)	0.0021 (4)	0.0037 (5)	0.0012 (4)
C3	0.0264 (6)	0.0227 (6)	0.0222 (6)	0.0016 (5)	0.0014 (5)	0.0014 (5)
C4	0.0308 (7)	0.0289 (7)	0.0232 (6)	0.0030 (5)	0.0036 (5)	0.0043 (5)
C5	0.0358 (7)	0.0296 (7)	0.0243 (6)	0.0027 (5)	−0.0012 (5)	0.0065 (5)
C6	0.0301 (7)	0.0295 (7)	0.0317 (7)	0.0061 (5)	−0.0029 (5)	0.0056 (5)
C7	0.0274 (7)	0.0318 (7)	0.0299 (6)	0.0068 (5)	0.0031 (5)	0.0030 (5)
C8	0.0274 (6)	0.0240 (6)	0.0211 (6)	0.0015 (5)	0.0011 (5)	0.0029 (4)
C9	0.0244 (6)	0.0246 (6)	0.0215 (6)	0.0022 (4)	0.0020 (5)	0.0020 (4)
C10	0.0250 (6)	0.0239 (6)	0.0200 (6)	0.0026 (4)	0.0043 (5)	0.0022 (4)
C11	0.0297 (6)	0.0299 (7)	0.0156 (5)	0.0065 (5)	0.0048 (4)	0.0038 (4)
C12	0.0299 (7)	0.0364 (8)	0.0307 (7)	0.0051 (5)	0.0025 (5)	0.0025 (5)
C13	0.0423 (8)	0.0360 (8)	0.0409 (8)	−0.0017 (6)	0.0064 (6)	0.0015 (6)
C14	0.0562 (9)	0.0290 (7)	0.0345 (7)	0.0098 (6)	0.0128 (7)	0.0044 (5)
C15	0.0423 (8)	0.0398 (8)	0.0325 (7)	0.0181 (6)	0.0097 (6)	0.0079 (6)
C16	0.0292 (7)	0.0354 (7)	0.0272 (6)	0.0086 (5)	0.0051 (5)	0.0054 (5)

### Geometric parameters (Å, º)

**Table:**

O1—C9	1.3687 (14)	C5—C6	1.3921 (19)
O1—C8	1.3774 (14)	C5—H5	0.9500
O2—C9	1.2158 (15)	C6—C7	1.3854 (18)
O3—C10	1.2245 (15)	C6—H6	0.9500
N1—C10	1.3528 (15)	C7—C8	1.3874 (18)
N1—N2	1.4064 (14)	C7—H7	0.9500
N1—H1N	0.896 (18)	C11—C12	1.3899 (19)
N2—C11	1.4184 (16)	C11—C16	1.3939 (17)
N2—H2N	0.911 (17)	C12—C13	1.388 (2)
C1—C2	1.3573 (16)	C12—H12	0.9500
C1—C9	1.4581 (17)	C13—C14	1.378 (2)
C1—C10	1.5027 (16)	C13—H13	0.9500
C2—C3	1.4315 (17)	C14—C15	1.384 (2)
C2—H2	0.9500	C14—H14	0.9500
C3—C8	1.3915 (18)	C15—C16	1.382 (2)
C3—C4	1.4064 (17)	C15—H15	0.9500
C4—C5	1.3809 (18)	C16—H16	0.9500
C4—H4	0.9500		
			
C9—O1—C8	122.88 (10)	O1—C8—C7	116.96 (11)
C10—N1—N2	120.49 (10)	O1—C8—C3	120.67 (11)
C10—N1—H1N	123.5 (11)	C7—C8—C3	122.37 (11)
N2—N1—H1N	116.0 (11)	O2—C9—O1	115.97 (10)
N1—N2—C11	115.52 (10)	O2—C9—C1	126.82 (11)
N1—N2—H2N	109.7 (10)	O1—C9—C1	117.20 (10)
C11—N2—H2N	112.6 (10)	O3—C10—N1	122.64 (11)
C2—C1—C9	119.98 (11)	O3—C10—C1	120.63 (11)
C2—C1—C10	117.83 (11)	N1—C10—C1	116.64 (10)
C9—C1—C10	122.18 (10)	C12—C11—C16	119.18 (12)
C1—C2—C3	121.33 (11)	C12—C11—N2	121.77 (11)
C1—C2—H2	119.3	C16—C11—N2	118.90 (12)
C3—C2—H2	119.3	C13—C12—C11	119.95 (13)
C8—C3—C4	118.47 (11)	C13—C12—H12	120.0
C8—C3—C2	117.82 (11)	C11—C12—H12	120.0
C4—C3—C2	123.71 (11)	C14—C13—C12	120.85 (14)
C5—C4—C3	119.62 (12)	C14—C13—H13	119.6
C5—C4—H4	120.2	C12—C13—H13	119.6
C3—C4—H4	120.2	C13—C14—C15	119.14 (13)
C4—C5—C6	120.62 (12)	C13—C14—H14	120.4
C4—C5—H5	119.7	C15—C14—H14	120.4
C6—C5—H5	119.7	C16—C15—C14	120.81 (13)
C7—C6—C5	120.82 (12)	C16—C15—H15	119.6
C7—C6—H6	119.6	C14—C15—H15	119.6
C5—C6—H6	119.6	C15—C16—C11	120.04 (13)
C6—C7—C8	118.10 (12)	C15—C16—H16	120.0
C6—C7—H7	121.0	C11—C16—H16	120.0
C8—C7—H7	121.0		
			
C10—N1—N2—C11	74.99 (14)	C2—C1—C9—O2	−175.93 (12)
C9—C1—C2—C3	−2.32 (18)	C10—C1—C9—O2	3.5 (2)
C10—C1—C2—C3	178.22 (10)	C2—C1—C9—O1	3.79 (17)
C1—C2—C3—C8	0.51 (18)	C10—C1—C9—O1	−176.78 (10)
C1—C2—C3—C4	−178.72 (11)	N2—N1—C10—O3	−0.14 (19)
C8—C3—C4—C5	0.41 (19)	N2—N1—C10—C1	176.63 (10)
C2—C3—C4—C5	179.64 (12)	C2—C1—C10—O3	11.13 (18)
C3—C4—C5—C6	0.6 (2)	C9—C1—C10—O3	−168.32 (11)
C4—C5—C6—C7	−1.1 (2)	C2—C1—C10—N1	−165.71 (11)
C5—C6—C7—C8	0.6 (2)	C9—C1—C10—N1	14.85 (17)
C9—O1—C8—C7	−178.13 (11)	N1—N2—C11—C12	28.34 (15)
C9—O1—C8—C3	1.88 (18)	N1—N2—C11—C16	−156.03 (11)
C6—C7—C8—O1	−179.58 (11)	C16—C11—C12—C13	1.27 (19)
C6—C7—C8—C3	0.4 (2)	N2—C11—C12—C13	176.89 (11)
C4—C3—C8—O1	179.08 (11)	C11—C12—C13—C14	−0.3 (2)
C2—C3—C8—O1	−0.19 (18)	C12—C13—C14—C15	−0.5 (2)
C4—C3—C8—C7	−0.91 (19)	C13—C14—C15—C16	0.3 (2)
C2—C3—C8—C7	179.82 (11)	C14—C15—C16—C11	0.7 (2)
C8—O1—C9—O2	176.16 (10)	C12—C11—C16—C15	−1.48 (18)
C8—O1—C9—C1	−3.59 (17)	N2—C11—C16—C15	−177.22 (11)

### Hydrogen-bond geometry (Å, º)

**Table:**

*D*—H···*A*	*D*—H	H···*A*	*D*···*A*	*D*—H···*A*
N1—H1*N*···N2^i^	0.896 (18)	2.327 (18)	3.0498 (14)	137.7 (15)
N1—H1*N*···O2	0.896 (18)	2.112 (18)	2.7544 (13)	127.8 (15)
N2—H2*N*···O2^ii^	0.911 (17)	2.243 (17)	3.1358 (14)	166.3 (14)

Symmetry codes: (i) −*x*+1, −*y*+1, −*z*; (ii) *x*−1, *y*, *z*.
