# Supplementary figures and images for: Crystal structure of 2-oxo-N′-phenyl-2H-chromene-3-carbohydrazide
Source: Acta Crystallogr E Crystallogr Commun. 2015 Nov 28;71(Pt 12):o1005–6. doi: 10.1107/S2056989015022495 (PMC4719947; doi:10.1107/S2056989015022495)

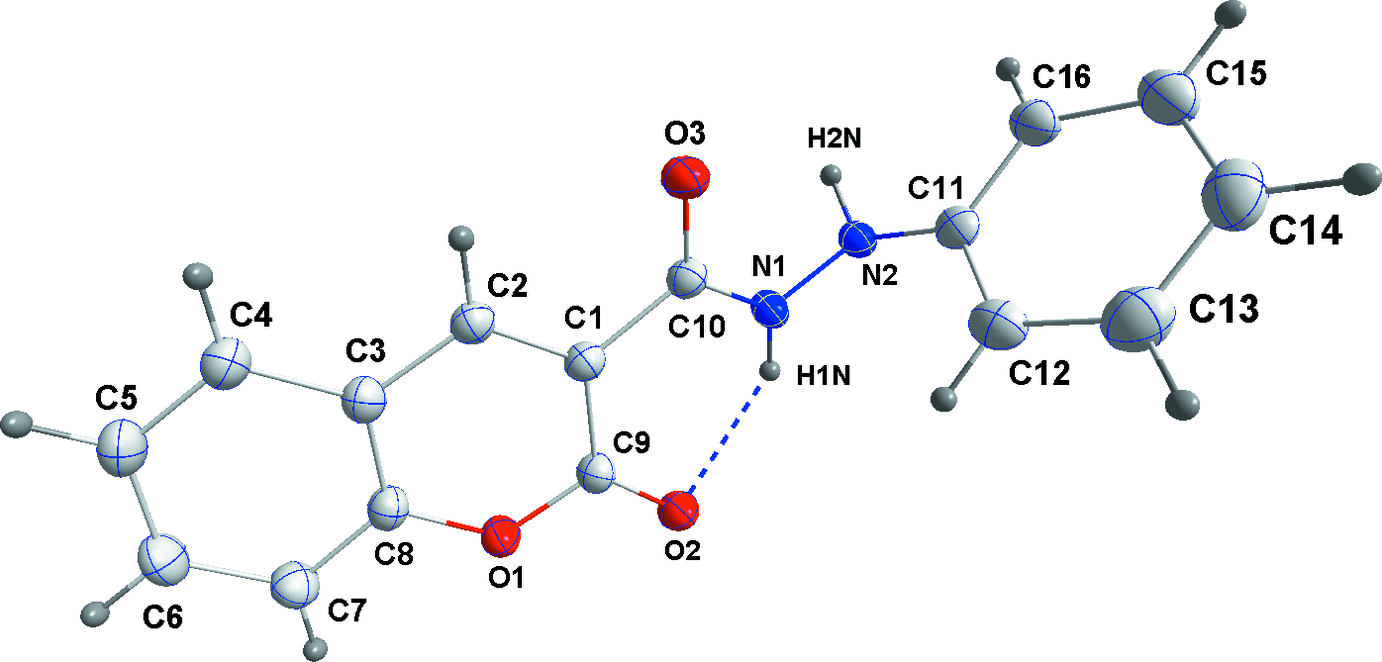

Supplement: Supplementary file 4 [file e-71-o1005-fig1.tif]

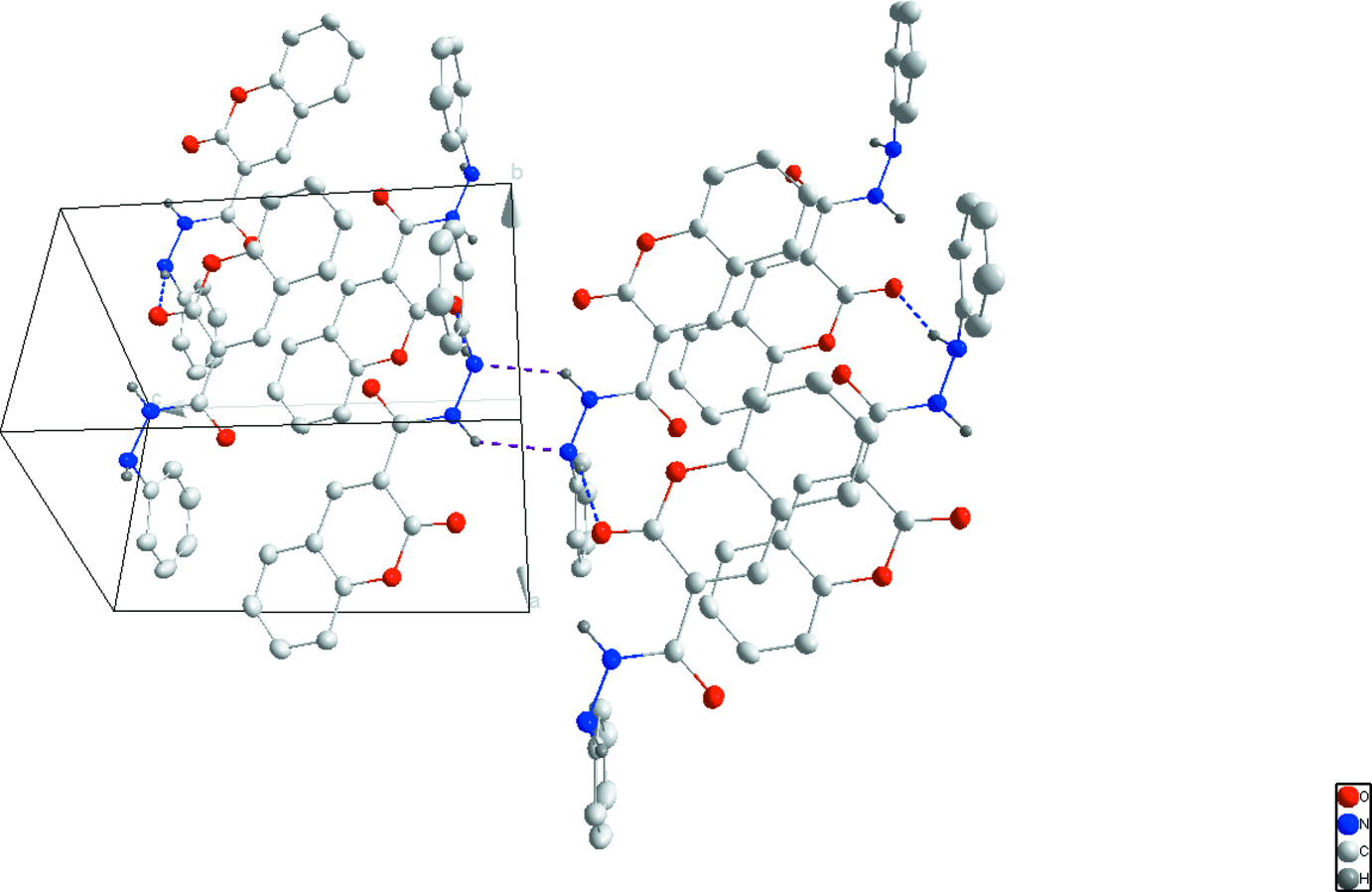

Supplement: Supplementary file 5 [file e-71-o1005-fig2.tif]
